# The RNA-binding protein Sam68 regulates expression and transcription function of the androgen receptor splice variant AR-V7

**DOI:** 10.1038/srep13426

**Published:** 2015-08-27

**Authors:** Jacqueline Stockley, Elke Markert, Yan Zhou, Craig N. Robson, David J. Elliott, Johan Lindberg, Hing Y. Leung, Prabhakar Rajan

**Affiliations:** 1 Institute of Cancer Sciences, University of Glasgow, Glasgow, UK; 2 Cancer Research UK Beatson Institute, Glasgow, UK; 3 Northern Institute for Cancer Research, Newcastle University, Newcastle-upon-Tyne, UK; 4 Institute of Genetic Medicine, Newcastle University, Newcastle-upon-Tyne, UK; 5 Department of Molecular Epidemiology and Biostatistics, Karolinska Institutet, Stockholm, Sweden

## Abstract

Castration-resistant (CR) prostate cancer (PCa) partly arises due to persistence of androgen receptor (AR) transcriptional activity in the absence of cognate ligand. An emerging mechanism underlying the CRPCa phenotype and predicting response to therapy is the expression of the constitutively-active AR-V7 splice variant generated by *AR* cryptic exon 3b inclusion. Here, we explore the role of the RNA-binding protein (RBP) Sam68 (encoded by *KHDRBS1*), which is over-expressed in clinical PCa, on AR-V7 expression and transcription function. Using a minigene reporter, we show that Sam68 controls expression of exon 3b resulting in an increase in endogenous AR-V7 mRNA and protein expression in RNA-binding-dependent manner. We identify a novel protein-protein interaction between Sam68 and AR-V7 mediated by a common domain shared with full-length AR, and observe these proteins in the cell nucleoplasm. Using a luciferase reporter, we demonstrate that Sam68 co-activates ligand-independent AR-V7 transcriptional activity in an RNA-binding-independent manner, and controls expression of the endogenous AR-V7-specific gene target *UBE2C*. Our data suggest that Sam68 has separable effects on the regulation of AR-V7 expression and transcriptional activity, through its RNA-binding capacity. Sam68 and other RBPs may control expression of AR-V7 and other splice variants as well as their downstream functions in CRPCa.

Prostate cancer (PCa) onset and progression is driven by androgen steroid hormones binding to their cognate androgen receptor (AR) nuclear hormone receptor transcription factor. Androgen deprivation therapy (ADT) is the mainstay of treatment for locally-advanced/metastatic PCa^1^ and inactivates AR signaling for a period of time by suppressing gonadal androgen biosynthesis. However, PCa inevitably becomes castration-resistant (CRPCa) due to a number of molecular mechanisms^2^, including the presence of a ligand-independent AR transcriptional programme^3^.

An evolving mechanism for the persistence of AR signaling in CRPCa is the generation of constitutively-active AR variants sharing a common amine (N)-terminus domain (NTD) and DNA-binding domain (DBD) but lacking a ligand-binding domain (LBD)^4^. These include several variants generated through alternative pre-mRNA splicing resulting in inclusion of cryptic exons containing a premature termination codon (PTC) resulting in translation of a truncated AR protein^5^. Of the AR splice variants, AR-V7 mRNA (Ensembl: ENST00000504326), which is generated by cryptic exon 3b inclusion, is an important predictive biomarker of response to second-line endocrine therapy^6^.

Although ligand-activated AR can affect overall expression of both full-length AR and AR-V7^7^,^8^, there is only one report to date studying the mechanisms of AR-V7 mRNA splicing^8^. Therein, the *trans*-acting RNA-binding proteins (RBPs) SRSF1 (encoded by *SRSF1*) and U2AF65 (encoded by *U2AF2*) were found to be involved in an ADT-mediated increase in AR-V7 expression. We have previously identified the RBP Sam68 (encoded by *KHDRBS1*) as an AR-interacting protein partner, and co-regulator of AR-dependent transcription and splicing^9^. Here, we test the functions of Sam68 on AR-V7 expression and transcriptional activity.

## Results

### Sam68 regulates AR exon 3b, AR-V7 mRNA and protein expression

**Figure 1:**
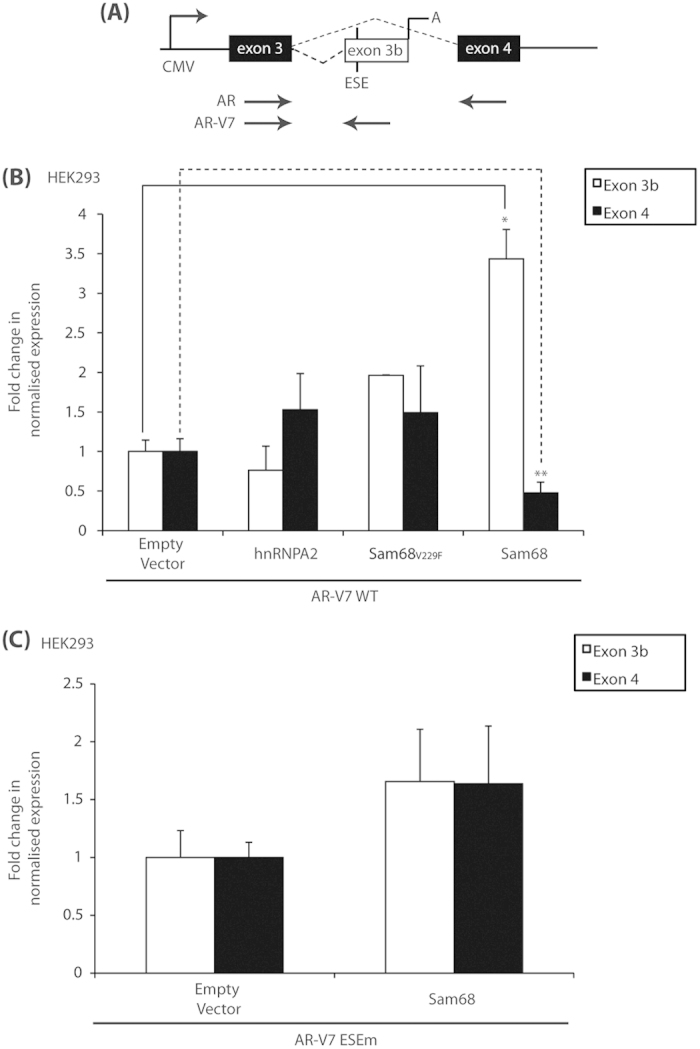
Sam68 regulates exon 3b expression. (**A**) AR-V7 minigene contains cryptic exon 3b with flanking constitutive exons 3 and 4 cloned downstream of a constitutively-active CMV promoter. Vertical line marks position of exonic splicing enhancer (ESE) near to the cryptic exon 3b 3′ splice site (SS). Arrows show location of primers used for qRT–PCR. (A = polyadenylation site). (**B**,**C**) HEK293 cells were cultured in full medium prior to transfection with the AR-V7 (B) or AR-V7 ESE mutant (ESEm) (**C**) minigenes (1.5 μg) with expression vectors for HA-hnRNPA2, GFP-Sam68_V229F_, GFP–Sam68 or empty vector control (500 ng) as indicated. qRT-PCR was performed on cDNAs and levels of exon 3b and exon 4 expression were normalised to *ACTB* levels and compared with empty vector control conditions to obtain the mean normalised fold-change in expression ± SE (^*^p = 0.003; ^**^p = 0.04).

Since Sam68 interacts with AR *in vitro* and co-activates AR-dependent transcription and splicing^9^, we hypothesised that Sam68 may also have an effect on the expression of transcripts encoding AR-V7. To test this hypothesis, we firstly used qRT-PCR to monitor expression of mRNAs containing *AR* cryptic exon 3b as well as constitutive exons 3 and 4 under the transcriptional control of the constitutively-active cytomegalovirus (CMV) promoter in a minigene reporter (Fig. 1A)^8^. HEK293 cells, which do not express endogenous AR protein, were cultured in full medium and transfected with the minigene and expression constructs encoding Sam68 protein, or a RNA-binding-deficient Sam68_V229F_ mutant^10^, or the Sam68-interacting RBP hnRNPA2 (encoded by *HNRNPA2B1*)^11^ as negative controls (Fig. 1B).

Ectopic expression of Sam68 protein resulted in a ~3.5-fold increase in cryptic exon 3b expression compared with the empty vector control (*p* = 0.0003) (Fig. 1B). Consistent with the preferential selection of exon 3b, Sam68 also caused ~50% reduction in exon 4 expression as compared with the empty vector control (*p* = 0.04). Ectopic expression of the Sam68-interacting hnRNPA2 protein did not have a statistically-significant effect on exon 3b (or exon 4) expression (*p* > 0.05), suggesting that exon 3b expression may be directly controlled by Sam68 itself rather than by other Sam68-interacting RBP partners.

Since Sam68-regulated exon inclusion is typically RNA-binding-dependent^10^, we employed the RNA-binding deficient Sam68_V229F_ mutant^10^ to determine whether the observed effects on cryptic exon 3b expression required the RNA-binding capacity of Sam68 protein. Compared with wild-type Sam68, the Sam68_V229F_ mutant was much less efficient at stimulating expression of exon 3b (~2-fold increase above controls), and did not have an effect on expression of mRNAs containing exon 4 (*p* > 0.05) (Fig. 1B).

Expression of the cryptic exon 3b has been show to be controlled, in part, by the RBP SRSF1 binding a *cis*-acting exonic splicing enhancer (ESE) close to the 3′ splice site (SS)^8^. Since Sam68 typically stimulates exon inclusion, we hypothesised that Sam68 may also regulate exon 3b expression through binding to the same ESE. To test this hypothesis, we employed the same minigene reporter but this time containing a point mutation within an ESE (ESEm) close to the 3′ SS (Fig. 1A), which has been shown to abrogate SRSF1-binding^8^. Despite the point mutation, ectopic expression of wild-type Sam68 resulted in a trend towards an increase in exon 3b expression as compared with the empty vector control (*p* = 0.14) (Fig. 1C). We also observed a similar Sam68-regulated increase in exon 4 expression.

**Figure 2:**
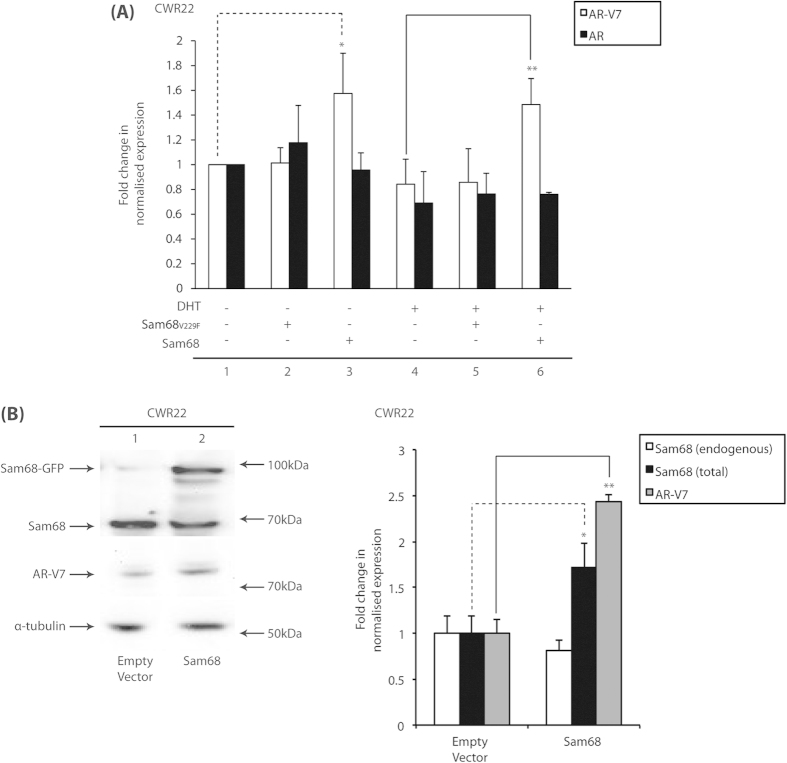
Sam68 regulates AR-V7 mRNA and protein expression (**A**) CWR22 cells were cultured in steroid-depleted medium with or without 100 nM DHT for 24 h prior to transfection with expression vectors for GFP-Sam68_V229F_ or GFP–Sam68 or empty vector control (2 μg) as indicated. qRT-PCR was performed on cDNAs and levels of AR and AR-V7 transcript expression were normalised to *ACTB* levels and compared with empty vector control conditions in the absence of DHT to obtain the mean normalised fold-change in expression ± SE (^*^p = 0.02; ^**^p = 0.002). (**B**) Representative Western blotting images of whole cell lysates from CWR22 cells cultured in full medium and transfected with the expression vector for GFP–Sam68 or empty vector (2 μg) as indicated (left panel). Densitometric band quantitation was performed to calculate the mean fold-change in endogenous Sam68, total (endogenous plus ectopic) Sam68, and AR-V7 protein expression ± SE compared to empty vector control conditions (^*^p = 0.03; ^**^p < 0.001).

To determine whether the Sam68-dependent increase in exon 3b expression translated into an increase in endogenous AR-V7 mRNA and protein expression *in vivo*, we employed the full-length AR- and AR-V7-expressing CWR22 and VCaP PCa cell lines^12^, which express *KHDRBS1* mRNA or Sam68 protein at similarly high levels to other common PCa cell lines (Supplementary Figure S1A and B). ADT/anti-androgen and androgen treatment have been shown to increase and decrease expression of AR-V7 protein, respectively, through possible recruitment of RBPs^8^,^13^. Hence, we used qRT-PCR to examine the effect of ectopic Sam68 protein expression and siRNA-mediated Sam68 knockdown on production of AR-V7 and full-length AR mRNA transcripts in CWR22 and VCaP cells grown in steroid-depleted medium as a model for ADT and in the presence of dihydrotestosterone (DHT) as the only available steroid (Fig. 2A, Supplementary Figure S1C and D).

In CWR22 cells, there was no statistically-significant difference in levels of both full-length AR and AR-V7 mRNAs between steroid-depleted and androgen-treated conditions (*p* > 0.05) (Fig. 2A, compare conditions 1 and 4). Ectopic expression of wild-type Sam68, but not the Sam68_V229F_ mutant, resulted in ~1.5-fold increase in AR-V7 mRNA expression both in the presence (*p* = 0.02) and absence of DHT (*p* = 0.002) (Fig. 2A, compare conditions 1 and 3, and 4 and 6). This increase in mRNA expression correlated with a ~2.4-fold Sam68-mediated increase in AR-V7 protein expression in cells cultured in full medium (*p* < 0.001) (Fig. 2B, right panel). However, there was no statistically-significant effect of ectopic Sam68 on full-length AR mRNA expression (*p* > 0.05) (Fig. 2A).

In the VCaP CRPCa cell line model, ectopic expression of wild-type Sam68 protein resulted in a ~1.5-fold increase in AR-V7 mRNA expression in the absence of DHT (*p* = 0.04) without an effect on full-length AR mRNA expression (*p* > 0.05) (Supplementary Figure S1C). However, siRNA-mediated depletion of Sam68 protein in CWR22 cells neither affected expression of AR-V7 nor full-length AR mRNAs in the presence or absence of DHT despite at least ~50% knockdown of Sam68 mRNA levels (Supplementary Figure S1D, compare lanes 1 and 2–4, and 5 and 6–8) which typically correlated with ~70% reduction in protein expression in cells cultured in full medium (Supplementary Figure S1E, compare lane 1 with 2–4).

### Sam68 and AR-V7 proteins interact and are present in the nucleoplasm of PCa cells

**Figure 3:**
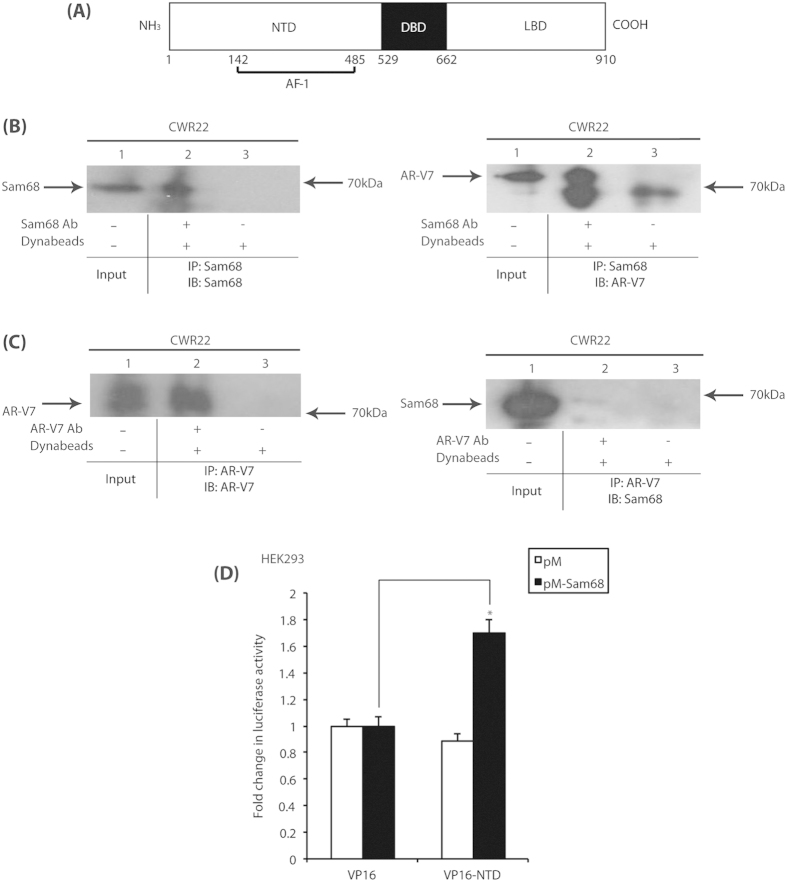
Sam68 and AR-V7 proteins interact via the AR NTD (**A**) AR comprises three functional domains: An amine-terminal transactivation domain (NTD), containing the activation function (AF) subdomain 1 (AF-1); a zinc finger-type DNA-binding domain (DBD); and a carboxyl-terminal ligand binding domain (LBD) containing AF subdomain 2 (AF-2). A hinge domain, containing a bipartite nuclear localisation signal (NLS) links the LBD and DBD. (**B**,**C**) Immunoprecipitation was performed in whole cell lysates from CWR22 cells cultured in full medium using either the antibodies to Sam68 (**B**) or AR-V7 (**C**), and recovered material was subjected to Western blotting as indicated. (**D**) HEK293 cells were cultured in full medium prior to transfection with UASTKLuc and pCMV-β-Gal reporters, and expression vectors for GAL4 DBD (pM; 100 ng) and VP16 AD (VP16; 100 ng) as indicated. After 48 hours, cells were harvested prior to analysis for luciferase and β-Galactosidase activities to provide relative luciferase activity, and compared with control conditions in the absence of vectors encoding AR NTD to obtain mean fold-change in luciferase activity ± SE (^*^p = 0.0001). (IP = immunoprecipitation; IB: Western immunoblot).

Consistent with published findings in LNCaP cells^9^, we identified a protein-protein interaction between Sam68 and full-length AR by immunoprecipitation in whole cell extracts from CWR22 cells cultured in full medium (Supplementary Figure S2A, right panel, compare lanes 1 and 2). Since CWR22 cells also express AR-V7 protein, we hypothesised that Sam68 may also interact with AR-V7, which contains the AR NTD but lacks the LBD required for ligand-dependent activity (Fig. 3A).

To test this hypothesis, both Sam68 and AR-V7 proteins were separately immunopreciptated in whole cell extracts from CWR22 cells cultured in full medium using their cognate antibodies prior to Western blotting (Fig. 3B,C). The antiserum to Sam68 efficiently immunoprecipitated Sam68 protein (Fig. 3B, left panel, compare lanes 1 and 2), and also co-immunoreprecipitated AR-V7 protein (Fig. 3B, right panel, compare lanes 1 and 2). Likewise, the antibody to AR-V7 efficiently immunoprecipitated AR-V7 protein (Fig. 2C, left panel, compare lanes 1 and 2), and also co-immunoreprecipitated Sam68 protein (Fig. 2C, right panel, compare lanes 1 and 2).

To verify this novel Sam68-AR-V7 protein-protein interaction, HEK293 cells cultured in full medium were transiently transfected with expression vectors for Sam68 and AR-V7 proteins (Supplementary Figure S2B). Consistent with the interactions between endogenous proteins in PCa cells, we also observed protein-protein interactions between ectopically-expressed Sam68 and AR-V7 proteins by immunoprecipitation (Supplementary Figure 2B, right panel, compare lanes 1 and 2). In all immunoprecipitation experiments, no or weak immunoprecipitation or co-immunoprecipitation was observed using normal mouse IgG (Supplementary Figure S2, all panels, compare lanes 2 and 3) or in the absence of either antibodies (Fig. 2B,C, all panels, compare lanes 2 and 3).

The above findings identify a novel protein-protein interaction between Sam68 and AR-V7. Since both full-length AR and AR-V7 share a common NTD (Fig. 3A), we hypothesised that Sam68 protein interacts with the AR NTD. To test this hypothesis, we used the mammalian 2-hybrid system. A VP16 activation domain (AD) fusion of AR NTD was co-expressed with a GAL4 DBD fusion of full-length Sam68 in HEK293 cells grown in the full medium (Fig. 3D). Ectopic expression of the VP16 AD-AR NTD fusion protein in the absence of the GAL4 DBD-Sam68 fusion protein did not affect reporter activity. However, in the presence of the GAL4 DBD-Sam68 fusion protein, ectopic expression of the AR NTD fusion protein resulted in ~1.7-fold increase in reporter activity (*p* = 0.0001).

Since Sam68 interacts with both full-length AR and AR-V7 proteins *in vivo*, we sought to determine if these proteins also co-localise *in vivo*. Using indirect immunofluorescence and confocal microscopy, we compared the intracellular distributions of endogenous Sam68 protein with full-length AR in LNCaP cells (Supplementary Figure S3). As previously-described^11^, Sam68 exhibited a diffuse nucleoplasmic distribution, and was concentrated in discrete Sam68/SLM nuclear bodies (SNBs) (Supplementary Figure S3, top left panel). AR predominantly exhibited a nucleoplasmic distribution, was excluded from the nucleolus and SC35-containing splicing speckles (Supplementary Figure S3, middle panels). Although both Sam68 and AR proteins were present in the nucleoplasm, they did not appear to co-localise to any discrete subnuclear organelle (Supplementary Figure S3, top right panel). In parallel experiments using the same combination of antibodies in PC3-M cells (which do not express endogenous AR protein) stably transfected with an expression vector for AR-V7, we observed similar localisation of Sam68 and AR-V7 proteins in the cell nucleoplasm (Supplementary Figure S3, bottom panels).

### Sam68 co-activates AR-V7 transcriptional activity and regulates expression of the AR-V7-specific target gene UBE2C

**Figure 4:**
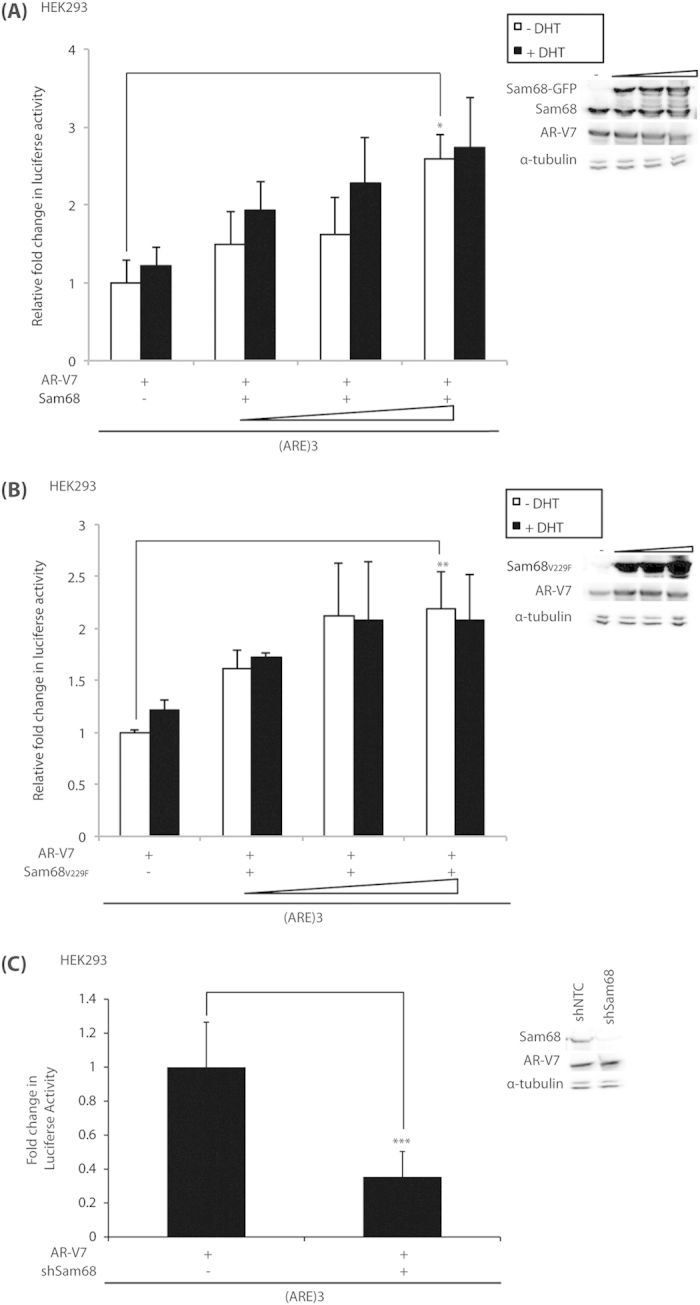
Sam68 co-activates AR-V7 transcriptional activity independent of RNA-binding capacity. (**A**–**C**) HEK293 cells were cultured in steroid-depleted medium with or without 100 nM DHT and transfected with the p(ARE)_3_Luc and pRL-null reporters, c-Flag AR-V7 (100 ng), together with expression vectors for (**A**) GFP-Sam68 or (**B**) GFP-Sam68_V229F_ or the empty vector control (50–200 ng) or (**C**) shRNA to Sam68 (shSam68) or the non-silencing control (shNTC) (200 ng) as indicated. After 24 hours, cells were harvested prior to analysis for luciferase and renilla activities to provide relative luciferase activity, and compared with control conditions in the absence of vectors encoding Sam68 (**A**,**B**) or shRNA to Sam68 (**C**) and DHT (**A**,**B**) to obtain mean fold-change in luciferase activity ± SE (^*^p = 0.01; ^**^p = 0.02; ^***^p = 0.03). Representative Western blots of whole cell lysates from HEK293 cells cultured in full medium and transfected with expression vectors for Sam68 (**A**,**B**) or shRNA to Sam68 (**C**) and AR-V7.

In light of the above and previously-published findings of a role for Sam68 on full-length AR-dependent transcriptional activity^9^, we hypothesised that Sam68 may also affect AR-V7-dependent transcription. Using an (ARE)_3_-driven luciferase reporter assay in HEK293 cells, we investigated the effects of ectopic expression and shRNA-mediated knockdown of Sam68 protein on AR-V7-dependent transcription (Fig. 4). The (ARE)_3_ reporter, which responds only to AR-binding, contains androgen response elements (AREs) from the core promoter region of the *KLK3* gene (see Materials and Methods). In keeping with published findings^9^, we observed that Sam68 protein functioned as a ligand-dependent co-activator of full-length AR-dependent transcriptional activity of the (ARE)_3_ reporter in an RNA-binding-independent manner (Supplementary Figure S4).

Consistent with the absence of the LBD, AR-V7-mediated reporter activity was not affected by the addition of DHT (Fig. 4A). In the presence and absence of DHT, AR-V7-mediated reporter activity was enhanced by increasing amounts of Sam68 (Fig. 4A). In the absence of DHT, we observed a maximal ~2.5-fold enhancement in reporter activity above the empty vector control (Fig. 4A) (p = 0.01). Consistent with our published data^9^, increasing amounts of RNA-binding-deficient mutant Sam68_V229F_ also enhanced AR-V7-mediated reporter activity in both the presence and absence of DHT (Fig. 4B). In the absence of DHT, we observed a maximal ~2.2-fold enhancement in reporter activity above the empty vector control (*p* = 0.02) (Fig. 4B). Ectopic expression of Sam68 protein did not alter (ARE)_3_ reporter activity in the absence of the AR (data not shown).

shRNA-mediated depletion of endogenous Sam68 protein also resulted in ~0.8-fold reduction in activity as compared with AR-V7-mediated reporter activity as compared with the level in the presence of Sam68 (*p* = 0.04) (Fig. 4C). Western blotting demonstrated that levels of AR-V7 or endogenous Sam68 protein expression were not typically affected by Sam68 protein overexpression or knockdown in cells cultured in full medium (Fig. 4, Western blots). Similar to published findings for full-length AR^9^, we observed maximal change in reporter activity between low and high Sam68 expression vector conditions, despite a less significant change in ectopic Sam68 protein expression levels.

**Figure 5:**
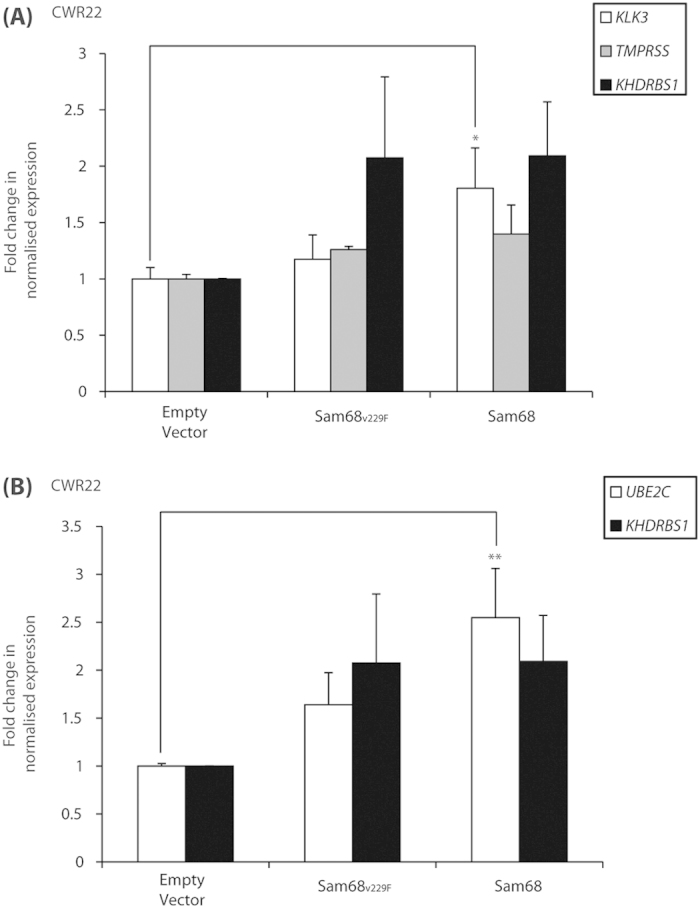
Sam68 regulates expression of the AR-V7-specific target gene *UBE2C.* (**A**,**B**) CWR22 cells were cultured in steroid-depleted medium for 24 h prior to transfection with expression vectors for GFP-Sam68_V229F_ or GFP–Sam68 or empty vector control (2 μg) as indicated. qRT-PCR was performed on cDNAs and levels of *KLK3*, *TMPRSS* (**A**) and *UBE2C* (**B**) were normalised to *ACTB* levels and compared with empty vector control conditions to obtain the mean normalised fold-change in expression ± SE (^*^p = 0.03; ^**^p = 0.02).

AR splice variants, including AR-V7, have been shown to regulate a transcriptional programme distinct to full-length AR in PCa cells^14^,^15^. Based on the above findings using the luciferase reporter assay, we hypothesised that Sam68 may also control expression of endogenous AR-V7 target genes *in vivo*. To test this hypothesis, we used qRT-PCR to examine the effect of ectopic Sam68 protein expression on canonical AR (*KLK3*, *TMPRSS*) or AR-V7-specific (*UBE2C*) target gene expression in CWR22 cells grown in steroid-depleted medium (Fig. 5). Ectopic expression of wild-type Sam68, but not the RNA-binding-deficient Sam68_V229F_ mutant, resulted in a ~1.8 fold increase in *KLK3* gene expression (*p* = 0.03) (Fig. 5A). However, neither wild-type Sam68 nor the Sam68_V229F_ mutant had a statistically-significant effect on *TMPRSS* gene expression (*p* > 0.05) (Fig. 5A). Ectopic expression of wild-type Sam68 resulted in ~2.5-fold increase in expression of the AR-V7-specific gene target *UBE2C* (*p* = 0.02) (Fig. 5B). We also observed a trend towards an increase in *UBE2C* gene expression following ectopic expression of the Sam68_V229F_ mutant (*p* = 0.06).

### Expression of the gene encoding Sam68 (KHDRBS1) is not altered in CRPCa

**Figure 6:**
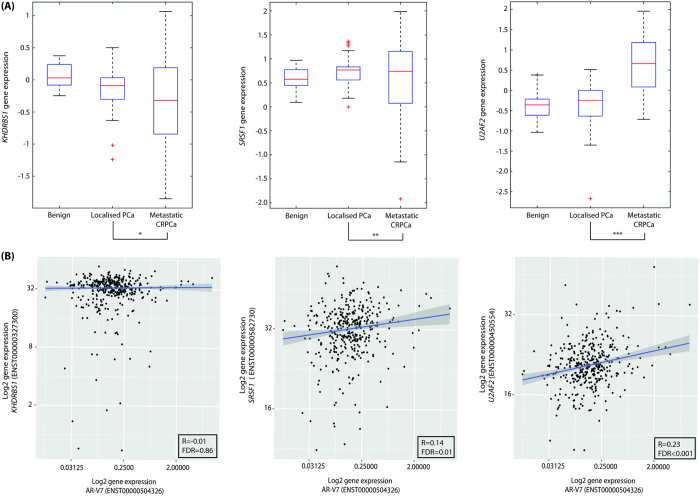
Expression of the gene encoding Sam68 (*KHDRBS1*) is not altered in CRPCa. (**A**) Expression of genes encoding Sam68 (*KHDRBS1*), U2AF65 (*U2AF2*), and SRSF1 (*SRSF1*) in benign prostate (n = 28), localized PCa (n = 59), and metastatic CRPCa (n = 35) from the Grasso dataset^18^. Gene expression is graphically represented using a box and whisker plot with outliers are indicated as +(^*^p = 0.19; ^**^p = 0.11; ^***^p < 0.001). (**B**) Scatter plots comparing expression of the major Ensembl transcripts encoding Sam68, SRSF1, and U2AF65 RBPs with AR-V7 from the TCGA dataset. A 95% confidence band is displayed for the regression lines.

Sam68 protein is highly expressed in common PCa cell lines including the CRPCa cell line models (Supplementary Figure S1A and B). Although Sam68 protein (encoded by *KHDRBS1*) has been shown to be over-expressed^9^,^16^ and phosphorylated^17^ in primary PCa, expression of Sam68 in CRPCa is unknown. We interrogated the Grasso dataset^18^ (n = 122) for changes in expression of *KHDRBS1* as well as *SRSF1* and *U2AF2*, which encode the RBPs SRSF1 and U2AF65, respectively, and have been implicated in AR-V7 splicing^8^ (Fig. 6A). There were no differences in *KHDRBS1* or *SRSF1* gene expression between primary localised PCa (n = 59) and CRPCa (n = 35) (*p* = 0.19 and *p* = 0.11, respectively) (Fig. 6A, left and middle panels). However, we observed a statistically-significant increase in *U2AF2* gene expression in CRPCa as compared with primary PCa (*p* < 0.001) (Fig. 6A, right panel).

To determine whether expression of genes encoding RBPs involved in AR-V7 expression (*KHDRB1, SRSF1*, and *U2AF*) correlated with expression of the AR-V7 mRNA in clinical PCa we used The Cancer Genome Atlas (TCGA) dataset (n = 417). We tested for correlations in expression of AR-V7 (ENST00000504326) and the major Ensembl transcripts encoding Sam68 (ENST00000327300), SRSF1 (ENST00000582730), and U2AF65 (ENST00000450554) (Fig. 6A). Although, there was no correlation between expression of the major transcript encoding Sam68 (n = 371; R = −0.01; FDR = 0.86) and AR-V7 (Fig. 6A, left panel), we did observe positive correlations in expression between AR-V7 and the major transcripts encoding SRSF1 (n = 371; R = 0.14; FDR = 0.01) and U2AF65 (n = 371; R = 0.23; FDR = 0.00003) (Fig. 6A, middle and right panels). Based on expression of these major Ensembl transcripts in the TCGA dataset, *KHDRBS1* (encoding Sam68) appeared to be expressed at higher levels than *U2AF2* and *SRSF1* in primary PCa (Fig. 6A, compare left panel with middle and right panels).

## Discussion

In this study, we demonstrate for the first time that the RBP Sam68 preferentially increases AR cryptic exon 3b expression, and controls mRNA and protein levels of the AR splice variant AR-V7 in PCa cells largely via an RNA-binding-dependent mechanism. Consistent with our previous findings for full-length AR^9^, we show that Sam68 and AR-V7 interact in PCa cells mediated by an association between Sam68 and the AR NTD. We show that Sam68 protein is present with full-length AR and AR-V7 in the cell nucleoplasm, where transcription and splicing occurs. We demonstrate that Sam68 functions as a co-activator of AR-V7-dependent transcriptional activity of the (ARE)_3_ reporter in an RNA-independent manner. Finally, we show that Sam68 controls expression of canonical AR and AR-V7 target genes in PCa cells via both RNA-binding-dependent and –independent mechanisms.

The emergence of constitutively-active AR splice variants in CRPCa, particularly AR-V7, poses a major clinical challenge as these variants not only seem to mediate resistance to novel inhibitors of androgen biosysthesis and ligand-dependent AR activity in PCa models^13^,^19^ but also appears to accurately predict response to treatment with these drugs in CRPCa patients^6^. To date, little is known of the mechanisms of generation of these splice variants and their downstream transcriptional targets. Moreover, it is unclear whether expression of AR splice variants in patients should preclude treatment with currently-available second-line endocrine agents.

Controlled by *trans*-acting complexes of several RBPs binding *cis*-acting regulatory pre-mRNA elements, alternative splicing can affect the processes underlying the hallmarks of cancer^20^ including PCa^21^. A potential *cis*-acting effect mediating AR-V7 expression may be due to an intragenic intron 1 deletion^22^, although exactly how this controls exon 3b inclusion is unclear. The *trans*-acting RBPs SRSF1 and U2AF65 appear to mediate an increase in AR-V7 expression in response to ADT *in vitro* through recruitment and binding to an intronic splicing enhancer (ISE) and ESE, respectively, close to the cryptic exon 3b 3′ SS^8^.

**Figure 7:**
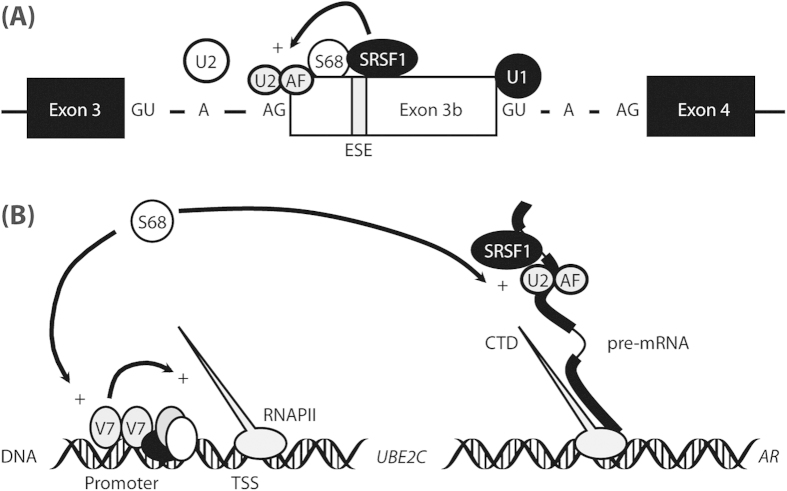
Model of Sam68-regulated AR-V7 splicing and downstream gene regulation. (**A**) AR cryptic exon 3b recognition is influenced by dinucleotides within splice site (SS) consensus sequences and auxiliary elements such as U2AF65 (U2AF) binding the 3′ SS; and the 5′ SS and branch point serve as binding sites for U1 and U2 snRNPs (U1 and U2), respectively. The exonic splicing enhancer (ESE) binds SRSF1 and Sam68 (S68) thereby recruit and stabilising binding of other spliceosome components such as U2AF65 to the 3′ SS. (**B**) Sam68 is recruited by the AR-V7 homodimer to the promoter region of target gene (e.g. *UBE2C*) and co-operates with other “general” transcription factors to enhance RNA polymerase (RNAPII)-mediated gene transcription from the transcription start site (TSS). Following activation (e.g. by protein phosphorylation), Sam68 is recruited to nascent pre-mRNA transcripts of target genes (e.g. *AR*) and in cooperation with other RNA-binding proteins (RBPs) regulates SS selection and exon inclusion.

Our data suggest that Sam68 also appears, at least in part, to have an effect on exon 3b expression via the SRSF1-responsive ESE (Fig. 7). This effect appears to be primarily dependent on its RNA-binding ability and not indirectly mediated by recruitment of Sam68-associated RBP complexes incorporating hnRNPA2^11^. The ability of the Sam68_V229F_ mutant to partially regulate exon 3b expression may be due to recruitment of endogenous wild-type protein, as Sam68 is thought to bind RNA as a homodimer^23^. Hence, the effect of Sam68 on exon 3b expression via the SRSF1-responsive ESE may be direct or, consistent with a role for Sam68 stabilising the *SRSF1* mRNA^24^, possibly indirectly mediated by SRSF1. In keeping with its role in U2AF65-mediated exon definition^25^, recruitment of U2AF65 by Sam68 to exon 3b 3′ SS, is another possible mechanism for Sam68-mediated AR-V7 expression (Fig. 7).

However, the trend towards a Sam68-regulated increase in exon 3b expression from the ESEm AR-V7 minigene suggests the effect of Sam68 may not be completely mediated by the ESE. An alternative mechanism, in keeping with its ability to control transcript stability via 3′-untranslated region (UTR)-binding^24^, might be stabilisation of mRNAs containing exon 3b thereby increasing AR-V7 expression. Since siRNA-mediated depletion of Sam68 protein does not affect AR-V7 (or full-length AR) levels, there may be redundancy in thes above putative mechanisms of action which are substituted by SRSF1 and U2AF65 and other RBPs. These observations are in keeping with several RBPs acting both synergistically and competitively within larger protein complexes to regulate SS selection in a context-dependent manner^26^.

Functionally, RBP interactions with AR protein have been shown to both activate^9^ and inhibit^27^ AR-dependent transcriptional activity. Although the RNA-binding capacity of Sam68 is primarily required to regulate *AR-V7* mRNA expression, our data suggest that this function is not required to co-activate AR-V7-dependent transcriptional activity. However, high total levels of Sam68 protein may be required for it to function as a co-activator of AR-V7-dependent transcriptional activity of the luciferase reporter (Fig. 4). Since both ectopic expression and shRNA-depletion of Sam68 protein affected AR-V7 dependent reporter activity, the effect of Sam68 on AR-V7-dependent transcription may be an important and non-redundant mechanism (Fig. 7). Consistent with this, we observed a trend towards an increase in expression of AR-V7-specific gene target *UBE2C* expression following ectopic expression of the transcriptionally-active but RNA-binding-deficient Sam68_V229F_ mutant. Taken together, these new data are in keeping with our previous findings^9^, which suggest that RNA processing and regulation of gene expression by Sam68 may be separable functions.

By knowledge-based validation, we were unable to identify up-regulation of *KHDRBS1* (encoding Sam68) gene expression in CRPCa and a correlation with AR-V7 expression in primary PCa, although we did reveal up-regulation of *U2AF*2 gene expression in CRPCa and correlations between *SRSF1* (encoding SRSF1) or *U2AF2* (encoding U2AF65) and AR-V7 mRNA expression in primary PCa. In the absence of any existing large datasets from CRPCa patients that allow similar transcript correlations, our observations in localised PCa suggest that absolute expression levels of *U2AF2* and possibly *SRSF1* may be important for regulation of AR-V7 mRNA expression in CRPCa. Since *KHDRBS1* is already expressed at higher levels in primary PCa as compared with *SRSF1* and *U2AF2*, post-translational modifications of Sam68 protein, rather than absolute expression levels, may be of greater importance for AR-V7 expression. Phosphorylation of Sam68 has been reported in primary PCa^17^, and is required for signal-dependent splicing regulation^28^.

Since AR-V7 protein appears to be able to bind to and regulate a distinct subset of genes in CRPCa cell line models^14^,^15^ and is an important predictive biomarker in clinical CRPCa^6^, a greater understanding of the mechanisms of AR-V7 expression and downstream functions will reveal a clearer role for AR-V7 (and other variants) in clinical CRPCa. Although *cis*-elements and other *trans*-acting RBPs (e.g. SRSF1 and U2AF65) appear to be involved, both directly and indirectly, Sam68 is the only RBP known to regulate AR-V7 expression and downstream transcription function that is over-expressed^9^,^29^ and phosphorylated^17^ in PCa. Hence overexpression and, moreover, activation of Sam68 protein through post-translational modification may contribute to the clinical CRPCa phenotype.

## Methods

### Antibodies, plasmids and oligonucleotides

The following antibodies were used: anti-Sam68 (sc333, Santa Cruz Biotechnology), anti-AR (G122-434) (BDB554225, BD Biosciences), anti-AR (N-20) (sc-816, Santa Cruz Biotechnology), anti-AR-V7 (AG10008, Precision Antibody), anti-SC35 (S4045, Sigma), anti-α-tubulin (TU-02, Santa Cruz Biotechnology), normal mouse IgG (sc-2025, Santa Cruz Biotechnology), anti-mouse IgG HRP-linked (7076, Cell Signaling), anti-rabbit IgG HRP-linked (7074, Cell Signaling), Alexa Fluor® 488 anti-Mouse IgG (A-21203, Life Technologies) and Alexa Fluor® 555 anti-Rabbit IgG (A-21429, Life Technologies). The following plasmids have been described previously or are commercially-available: pEGFP3-Sam68^30^; pEGFP-Sam68_V229F_^10^; pcDNA3-HA-hnRNPA2-cDNA^31^; pcDNA3-AR, UASTKLuc, and pCMV-β-Gal^32^; AR-V7 minigenes^8^; pLKO-si-Sam68^29^; and control pLKO-puro Non-Target shRNA Control (SHC016, Sigma) and pRL-null (E2271, Promega). pM-Sam68 was generated by cloning of the Sam68 coding sequence from pGBTK7-Sam68^33^ into the empty pM vector between *Eco*RI and *Sal*I. cFlag AR-V7 was generated by cloning of a PCR-amplified cDNA fragment containing the AR-V7 coding sequence from the 22Rv1 PCa cell line (CRL-2505, ATCC) into c-Flag pcDNA3^34^ between *Xho*I and *Bam*HI. p(ARE)_3_Luc contains 3x repeats of the 1st androgen response element (ARE) plus TATA box from *KLK3* promoter region cloned into pGL3basic (E1751, Promega). The identity and integrity of newly-generated clones was confirmed by Sanger sequencing. Sequences used to generate siRNA duplexes are as previously described^35^, and a non- silencing control (#D-001810-01-20, Dharmacon) was also used. Sequences used to generate oligonucleotide primers for PCR were as previously described^8^,^36^ or are listed in Supplementary Table S1.

### Cell Culture, DNA and RNA transfections

All cells were grown at 37 °C in 5% CO_2_. HEK293 (CRL-1573, ATCC), 22Rv1 (CRL-2505, ATCC), VCaP (CRL-2876, ATCC) and CWR22^37^ cells were maintained in RPMI-1640 medium (31870-025, Life Technologies) with 2 mM L-glutamine (25030-024, Life Technologies), supplemented with 10% foetal bovine serum (FBS) (A15-101, PAA Laboratories) or 10% Dextran Charcoal Stripped FCS (12676011, Life Technologies) to produce steroid-depleted medium as detailed in figure legends. PC3-M cells were derived as previously described^38^ and maintained as above. PC3-M cells stably-expressing AR-V7 were generated by transfection of parental PC-3M cells with the c-Flag AR-V7 vector. Cells were cultured in medium containing 300 μg/ml G418S (G418S, Formedium), and resistant clones were maintained under selection but removed for experiments. Transfections with plasmid DNA and siRNA duplexes were carried out as detailed in the figure legends using Lipofectamine LTX (15338-100, Life Technologies) and RNAiMax (13778-075, Life Technologies), respectively, according to manufacturer’s instructions. Where indicated, cells were treated with 100 nM dihydrotestosterone (DHT).

### Immunoprecipitation, SDS-PAGE, and Western blotting

Protein immunoprecipitation was performed as previously described^39^. Recovered material or whole cell lysates were resolved by SDS-PAGE and subjected to Western blotting as previously described^36^. Antibody concentrations were as follows: anti-Sam68 (1:4000), anti-AR-V7 (1:1000), anti-AR (N-20) (1:1000), anti-α-tubulin (1:1000); HRP-linked secondaries (1:2000). Immunoprecipitation data shown are representative of two independent biological experimental replicates. Where indicated, densitometric assessments of protein bands were performed using ImageJ (http://rsb.info.nih.gov/ij/), and intensities used to calculate relative normalised fold-change in protein expression. Western blotting data shown are from at least two independent biological experimental replicates.

### Indirect immunofluorescence microscopy

LNCaP and PC3-M cells were grown and transfected on glass coverslips (VWR International), and stained with primary and secondary antibodies as previously described^36^. Antibody concentrations were as follows: anti-Sam68 (1:1000), anti-AR (G122-434;1:20) (N-20; 1:100), anti-SC35 (1:200); Alexa Fluor-linked secondaries (1:400). Images shown are representative of at least two independent biological experimental replicates.

### RNA extraction, reverse transcription (RT) and quantitative PCR (PCR)

Total RNA extraction, quantitative RT-PCR (qRT-PCR), and relative gene expression analyses were performed as previously described^36^. Data shown are from three independent biological experimental replicates with three technical replicates.

### Minigene Splicing Assays

HEK293 cells were seeded at a density of 1 × 10^5^ cells/ml in full medium into 6-well plates (Asahi Techno Glass). Cells were transfected with DNA as detailed in figure legends. RNA was extracted using RNeasy (Qiagen) and subjected to qRT-PCR using oligonucleotides as previously described^8^. Data shown are from four independent biological experimental replicates with three technical replicates.

### Luciferase Reporter Assays

For mammalian 2-hybrid assays, HEK293 cells were seeded at a density of 5 × 10^4^ cells/ml in full medium into 12-well plates (Asahi Techno Glass). Cells were transfected with DNA as detailed in the figure legends. Luciferase and β–Galactosidase assays were performed to give relative activity as previously described^32^. Data shown are from at least two independent biological experimental replicates with four technical replicates. For transcription reporter assays, HEK293 cells were seeded at a density of 2 × 10^4^ cells/well in steroid-depleted medium into 24-well plates (Asahi Techno Glass). Cells were transfected with DNA, and supplemented with 100 nM DHT as detailed in figure legends. Firefly and Renilla luciferase assays were carried out using the Dual Luciferase Reporter Assay system (E1910, Promega) as per manufacturer’s instructions to give relative luciferase activity. Data shown are from at least three independent biological experimental replicates with three technical replicates.

### Bioinformatics

Pre-processed microarray data were downloaded from the National Center for Biotechnology Information (NCBI) Gene Expression Omnibus (GEO)^40^. For cell lines, gene expression data were extracted from the Taylor dataset^41^ (Accession number: GSE21034), normalized as the mean of the log_2_ transformed probe signal, and plotted against the PCa cell lines as annotated in the sample information. For clinical samples, median-centered gene expression data were extracted from the Grasso dataset^18^ (Accession number: GSE35988), normalized as the mean of the array probes, and plotted against clinical groups (benign prostate, localized PCa and metastatic CRPCa) as annotated in the sample information. Analysis of Variance (ANOVA) was employed to test for differences in means between groups with *p* < 0.05 taken to indicate statistical significance (MATLAB, MathWorks). FASTQ files containing pre-processed (Level 3) RNA-Seq data were downloaded from The Cancer Genome Atlas (TCGA) data portal (Accession number: 23700-2). Adaptors were trimmed using Skewer^42^ and isoform quantification performed using Sailfish^43^. The R software v.3.1.2^44^ MASS (Modern Applied Statistics with S) package^45^ was used for robust linear modeling. Spearman correlation was performed on data from samples harboring non-zero expression in both genes of interest and *q*-values reported using the false discovery rate (FDR) with FDR < 0.05 taken to indicate statistical significance.

### Statistical analyses

Graphical data shown represent the means ± standard error (SE) of independent experiments. The one-tailed independent sample T-Test was employed to identify differences in means between groups with *p* < 0.05 taken to indicate statistical significance.

## Additional Information

**How to cite this article**: Stockley, J. *et al.* The RNA-binding protein Sam68 regulates expression and transcription function of the androgen receptor splice variant AR-V7. *Sci. Rep.* **5**, 13426; doi: 10.1038/srep13426 (2015).

## Supplementary Material

Supplementary Information
