# Supplementary material for: The RNA-binding protein Sam68 regulates expression and transcription function of the androgen receptor splice variant AR-V7
Source: Sci Rep. 2015 Aug 27;5:13426. doi: 10.1038/srep13426 (PMC4550848; doi:10.1038/srep13426)
Supplement: Supplementary Information [file srep13426-s1.pdf]

Supplementary Information

The RNA-binding protein Sam68 regulates expression and transcription function of the androgen receptor splice variant AR-V7

Jacqueline Stockley, Elke Markert, Yan Zhou, Craig N. Robson, David J. Elliott, Johan Lindberg, Hing Y. Leung, and Prabhakar Rajan

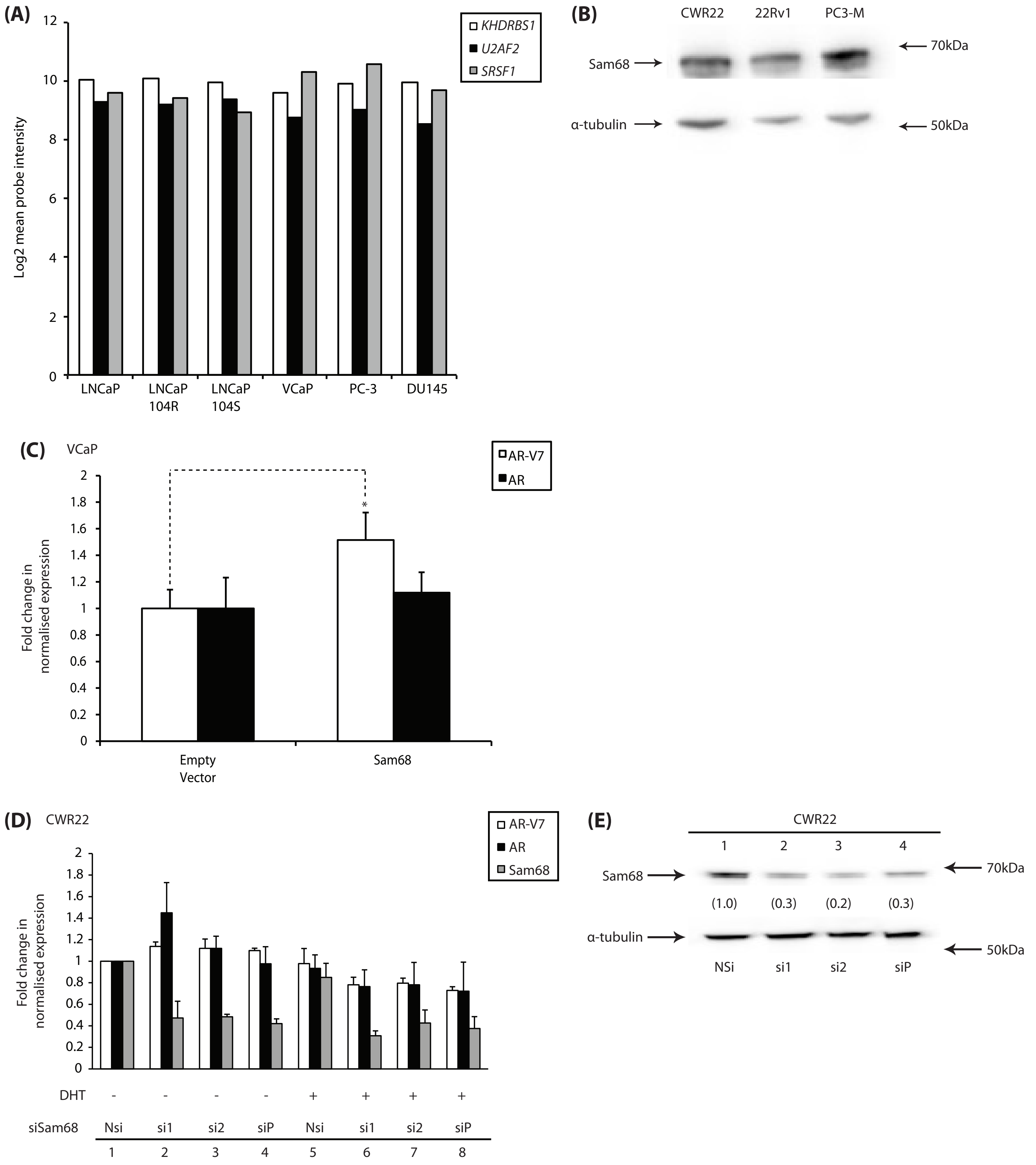

Supplementary Figure S1: Expression and effect of Sam68 on AR-V7 mRNA in PCa cell lines. (A) Expression profiles of genes encoding Sam68 (KHDRBS1), U2AF65 (U2AF), and SRSF1 (SRSF1) in PCa cell lines in the Taylor dataset 41. (B) Representative Western blots of whole cell lysates from PCa cell lines in steady-state conditions. (C) VCaP cells were cultured in steroid-depleted medium prior to transfection with expression vectors for GFP–Sam68 or empty vector control (2 μg) as indicated. qRT-PCR was performed on cDNAs and levels of AR and AR-V7 transcript expression were normalised to ACTB levels and compared with empty vector control conditions to obtain the mean normalised fold-change in expression ± SE. (*p=0.04). (D) CWR22 cells were cultured in steroid-depleted medium with or without 100 nM DHT for 24 h prior to transfection with two separate siRNA duplexes to Sam68 (si1 or si2), or a combination (siP), or non-silencing (Nsi) control to final concentration of 25 nM. qRT-PCR was performed on cDNAs and levels of AR and AR-V7 expression were normalised to ACTB levels and compared with Nsi control conditions in the absence of DHT to obtain the mean normalised fold-change in expression ± SE. (E) Representative Western blots of whole cell lysates from CWR22 cells cultured in full medium and transfected with siRNA duplexes. Densitometric band quantitation was performed to calculate the relative normalised fold-change in Sam68 protein expression compared with Nsi control conditions (shown in brackets).

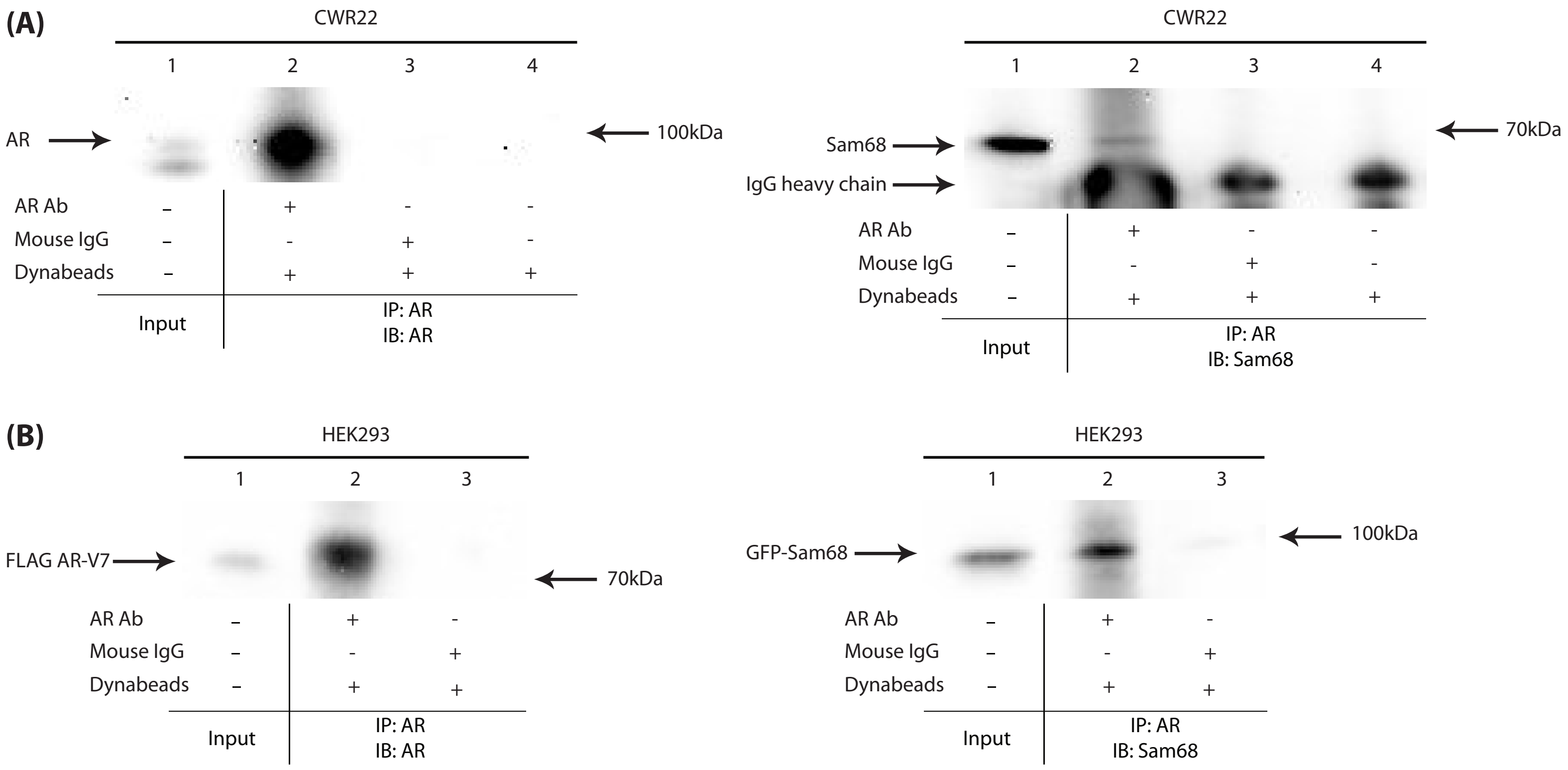

Supplementary Figure S2: Sam68 interacts with both full-length AR and AR-V7 proteins. (A) Immunoprecipitation was performed in whole cell lysates from CWR22 cells cultured in full medium using antibody to AR (N-20), and recovered material was subjected to Western blotting as indicated. (B) HEK293 cells were cultured in full medium prior to transfection with expression vectors for c-Flag-AR-V7 (1 µg) and GFP-Sam68 (1 µg). After 48 hours, cells were harvested and subjected to immmunoprecipitation using the antibody to AR (N-20), and recovered material was subjected to Western blotting as indicated. (IP=immunoprecipitation; IB: Western immunoblot).

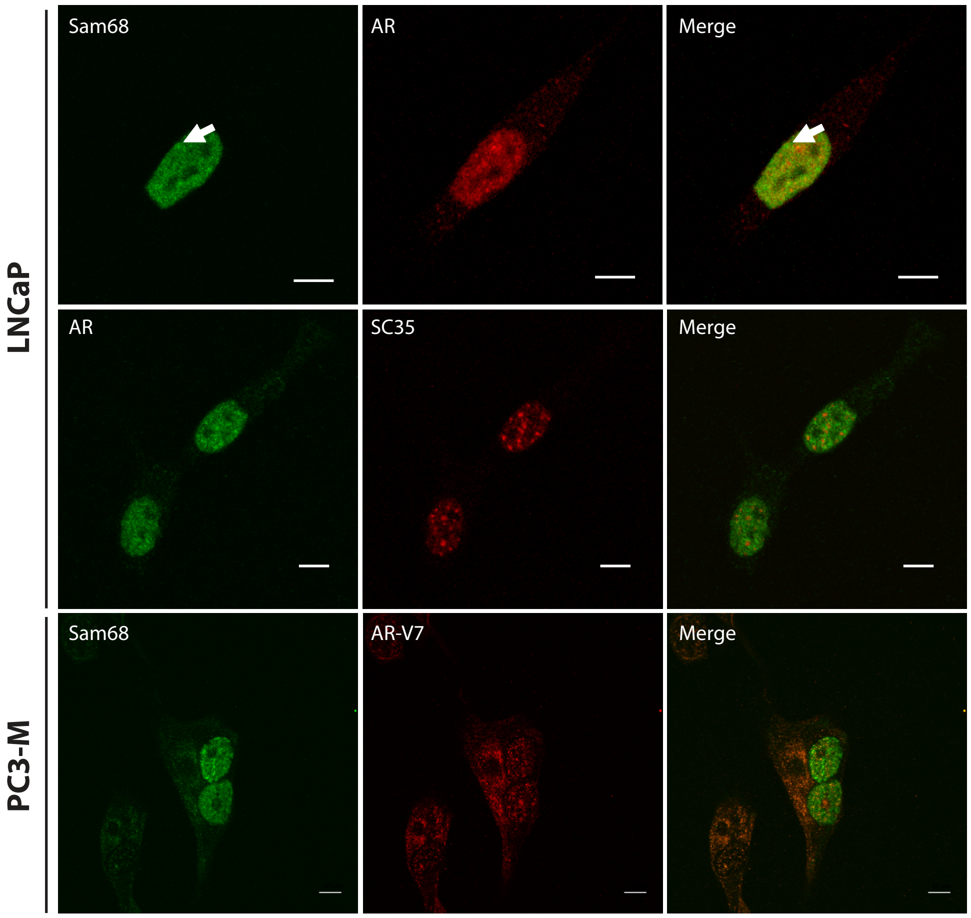

Supplementary Figure S3: Intracellular localisation of Sam68, full-length AR and AR-V7. Representative indirect immunofluorescence images of LNCaP or PC3-M stably transfected with the expression vector for cFlag-AR-V7. Images were captured by confocal laser scanning microscopy using antibodies to Sam68 (upper and lower panels), SC-35 (middle panel) and AR (all 3 panels). Sam68 protein was concentrated within SNBs (arrowed). (Bar=10 µm).

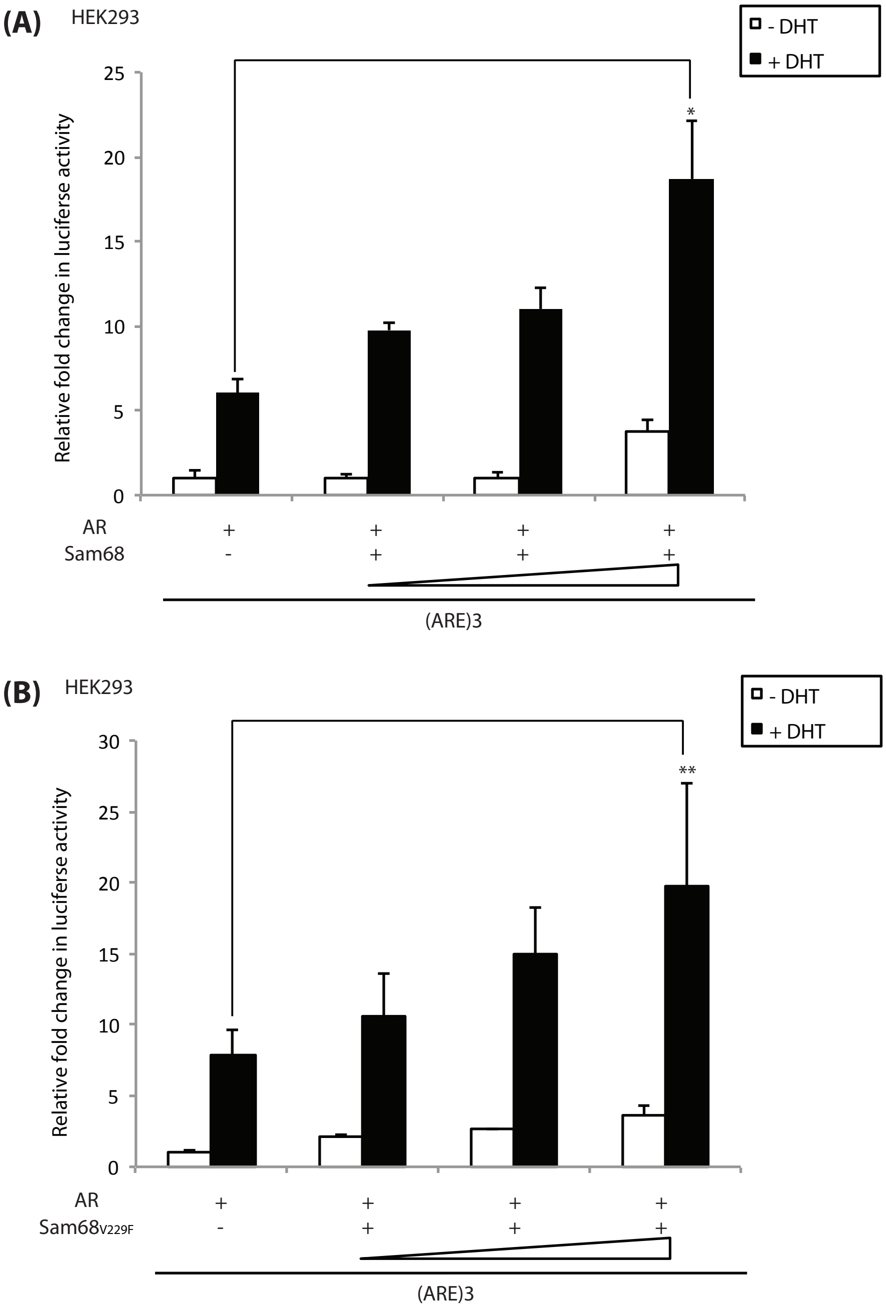

Supplementary Figure S4: Sam68 enhances AR-dependent transcription of a luciferase reporter independent of RNA-binding capacity. (A and B) HEK293 cells were cultured in steroid-depleted medium with or without 100 nM DHT and transfected with the p(ARE)3Luc and pRL-null reporters, AR (100 ng), together with expression vectors for (A) GFP-Sam68 or (B) GFP-Sam68$_{V229F}$ or the empty vector control (50-200 ng) as indicated. After 24 hours, cells were harvested prior to analysis for luciferase and renilla activities to provide relative luciferase activity, and compared with control conditions in the absence of vectors encoding Sam68 and DHT to obtain mean fold-change in luciferase activity ± SE. (*p=0.02; **p=0.06).

PCR type	Name	5'-3'	Primer	qRT-PCR method	Roche UPL probe number
**qRT-PCR**	AR-Pan-F	F	CCATCTTGTCGTCTTCGGAAATGTTATGAAGC	SYBR	N/A
	AR-FL-R	R	AGCTTCTGGGTTGTCTCCTCAGTGG	SYBR	N/A
	AR-V7-R	R	TTTGAATGAGGCAAGTCAGCCTTTCT	SYBR	N/A
	AR minigene-Exon3b F	F	CAGGGATGACTCTGGGAGAA	SYBR	N/A
	AR minigene-Exon3b R	R	GCCCTCTAGAGCCCTCATTT	SYBR	N/A
	AR minigene-Exon4-control F	F	TCTTGTCGTCTTCGGAAATGT	SYBR	N/A
	AR minigene-Exon4-control R	R	AAGCCTCTCCTTCCTCCTGTA	SYBR	N/A
	TMPRSS2	F	CAGGAGTGTACGGGAATGTGATGGT	SYBR	N/A
	TMPRSS2	R	GATTAGCCGTCTGCCCTCATTTGT	SYBR	N/A
	KHDBRS1	F	CTCCTGCTAGGCCAGTGAA	SYBR	N/A
	KHDRBS1	R	TTGTGGGTAAAGCAACAGGA	SYBR	N/A
	UBE2C	F	GGTGGGCAAAAGGCTACA	UPL	#17
	UBE2C	R	AATCCCTTTATCGCCAGACA	UPL	#17
	KLK3	F	CCTGTCCGTGACGTGGAT	UPL	#75
	KLK3	R	CAGGGTTGGGAATGCTTCT	UPL	#75
	ACTB	F	ATTGGCAATGAGCGGTTC	UPL	#11
	ACTB	R	CGTGGATGCCACAGGACT	UPL	#11
**gDNA PCR**	c-Flag AR-V7	F	CTAAGCAGCTCGAGTCCCGCAAGTTTCCTTCTCT	N/A	N/A
	c-Flag AR-V7	R	TGCTTAGCGGATCCGGGTCTGGTCATTTTGAGATGC	N/A	N/A

Supplementary Table S1: Primer sequences. Sequence of oligonucleotides used for PCR of genomic DNA (gDNA) and qRT-PCR of RNA with SYBR Green (11762100, Life Technologies) or Universal Probe Library (UPL) (Roche, 04683633001) with probe number.
